# Spreading the sharing economy: Institutional conditions for the international diffusion of Uber, 2010-2017

**DOI:** 10.1371/journal.pone.0248038

**Published:** 2021-03-09

**Authors:** Hyun J. Kim, Chan S. Suh

**Affiliations:** Department of Sociology, College of Social Sciences, Chung-Ang University, Seoul, South Korea; Institute for Advanced Sustainability Studies, GERMANY

## Abstract

This study examines the factors that facilitated the international diffusion of Uber, one of the fastest growing global companies in the sharing economy. We particularly focus on the legal and institutional conditions under which this ride-sharing platform could spread to customers online. Using a unique cross-national, longitudinal dataset, we employ event history models to investigate the effect of institutional environment on the diffusion of Uber. The results suggest that the establishment of the rule of law has a positive impact on the spread of Uber, even after controlling for economic and political characteristics. In addition, the overall quality of governmental regulations on markets is positively related to the diffusion of this ride-sharing platform. Our study contributes to the emerging literature on the sharing economy by identifying critical institutional factors that enable the transformation of business models worldwide.

## Introduction

Since the rise of the information and communications technology (ICT), software intermediaries such as digital platforms and applications have been developed as an emergent industry to facilitate hyper-connectivity among individuals. In this so called network society [1], the “sharing economy” has emerged as an alternative to the traditional economy to unlock the values of underutilized products and assets [2–4]. Scholars have suggested conceptual-theoretical frameworks to understand the dynamics as well as future potentials of the sharing economy [5, 6]. Herein, digital platforms have been identified as a social space to facilitate both social interactions and economic transactions. Within this new space, strangers can maximize their use-value without claiming one’s ownership over a wide form of commodities [7–9].

While previous studies on the sharing economy have mostly paid attention to the development of digital technology and the internal governance of sharing economy organizations, scholars have just started to illuminate the external factors that facilitate the dynamics of sharing economy [10]. Regarding the external environment, one major line of research has suggested the importance of economic and cultural environments in facilitating users’ participation in sharing economy [7, 11–14]. Another line of research has focused on the political environment by showing how local governance influences the expansion of sharing economy [15–19]. What is still underexamined is the institutional environment affecting the rise of the sharing economy at the international level. This is a serious oversight given that, firstly, the development of an economy depends heavily on institutions such as formal rules and regulations [20], and secondly, the global diffusion of sharing economy is better captured from an international perspective, not from a domestic approach.

To fill this gap in the nascent literature of sharing economy, we investigate how institutional conditions shape the diffusion of sharing economy platforms on a global scale. We particularly zoom in the case of Uber Tech. Inc., one of the most popular and fastest-growing sharing platform companies. To study the conditions that enabled the successful spread of this startup company, we employ event history modeling to analyze a unique cross-national, longitudinal dataset we collected on 142 countries over the period between 2010 and 2017. The results suggest that institutional factors such as the established rule of law and the quality of governmental regulations on markets play a critical role in facilitating the diffusion of Uber, even after controlling for other economic and political characteristics. Implications of our findings to the sharing economy industry and its scholarship are discussed in conclusion.

## Theoretical background

### The advent of the sharing economy

The rise of information technology in the late 20th century paved the way for the spread of integrating personal computers, mobile communication and ubiquitous computing, globally integrated financial market, virtual-reality convergence culture, and other unprecedented phenomenon. Castells [1] identified these dramatic changes we observe as the advent of the network society where ICT facilitates hyper-connectivity among people. As the logic of network propagates globally, the ICT paradigm has facilitated new forms of production, experience, power, culture, and such.

In this highly-connected society, digital platforms have emerged as a mediator for person-to-person sharing transactions and interactions. There is no commonly shared understanding about the notion of the sharing economy. Scholars admit that those are hard to be established due to its multifaceted aspects [3]. However, related to the concept of shareable goods, sharing economy can be viewed as the economy where “consumers granting each other temporary access to under-utilized physical assets, possibly for money” [5]. Herein, shareable goods with excess capacity are reproduced, where the owner does not need to occupy and consume them all the time [21]. As the catchphrase ‘access before ownership’ implies, participants in sharing economy platforms can maximize their use-value without claiming one’s ownership over commodities [5, 8].

It is hard to deny that societies have always shared goods and services [22]. What distinguishes the current modality from the past is “stranger sharing” [3]. The re-emergence of sharing has expanded its boundary from the private to the public realm, capturing scholars’ attention as a new form of economic production. Interpersonal sharing activities within digital platforms have made the transformation possible. This transformation gained its name as sharing economy, distinguished from the traditional economy [4, 9]. In principle, sharing economy pursuits alternative ownership over private ownership, resource-saving over resource-consuming, value creation over profit creation, and cooperation over competition [23]. Hence, sharing economy does not merely facilitates interpersonal transaction but also enables alternative governance wherein participants of a given platform share ownership [6, 24, 25]. In this new type of economy, digital transaction networks emerge as new social relationships with alternative ethics surrounding sharing interactions [26]. The “sharing of processing, storage and communication platforms” [22] has begun to transform the traditional protocols for exchanging goods and services [27].

To fully capture the dynamics revolving around sharing economy, it is crucial to understand both the internal/technological logic within platform operations and the external/institutional logic embedding platforms [10]. Compared to conceptual-theoretical studies focusing on the sharing governance and the potential impact of sharing economy models, however, empirical studies have paid less attention to the institutional environment enabling the diffusion of digital platforms worldwide [28].

### The environment of the sharing economy

The nascent literature on sharing economy has started to illuminate the external factors affecting the institutionalization of sharing economy. Bardhi and Eckhardt [7], for example, suggested from their study of Zipcar consumers that self-interests and utilitarian incentives are important to establish car sharing platforms. An exploratory research by Alonso-Almeida, Perramon, and Bagur-Femenías [11] has also examined how consumers’ changing attitude towards materialism influenced their level of participation in sharing economy. In addition, Barnes and Mattsson [12] illuminated the relationship between consumers’ participation in sharing economy platforms and their changing perception of access-based consumption in not just economic, but also environmental and social dimensions. Schor et al. [13] analyzed how social and economic inequality isreproduced within the sharing economy arrangements, focusing on the four sites of sharing platforms. In a similar vein, Wood et al. [14] shed light on both the relational embeddedness and normative disembeddedness of digital platforms from the preexisting social environment. Regarding political conditions, previous studies focused on the domestic political dynamics of local governance that shaped the adoption of sharing platforms [15–19].

Building upon these literature on the external environment surrounding sharing economy, we focus on the importance of formal rules and regulations or the institutional factors that are critical to the rise of the market economy [20]. Since the diffusion of the sharing economy in the network society is intrinsically a global phenomenon [28], we examine the legal and institutional environment affecting the rise of sharing platforms in a comparative, cross-national setting.

### The international expansion of Uber

Among various sharing platforms, Uber stands out as one of the most critical cases to study sharing economy. As a mobility sharing platform, Uber linked drivers and riders with lower prices, quicker matching, and a more convenient payment system compared to the conventional taxi industry [29]. Uber was nominated as one of the most valued startups as of 2018. It is true that the rise of Uber was not celebrated unanimously by researchers in the domain. The Silicon Valley’s success such as Uber and Airbnb was criticized as not being qualitatively different from traditional types of firms for due to their profit-driven orientation and centralized operation practices [30–32]. But still, it is hard to deny the critical role of Uber in popularizing the alternative form of economic relation labeled as ‘sharing’ [5, 25].

**Fig 1:**
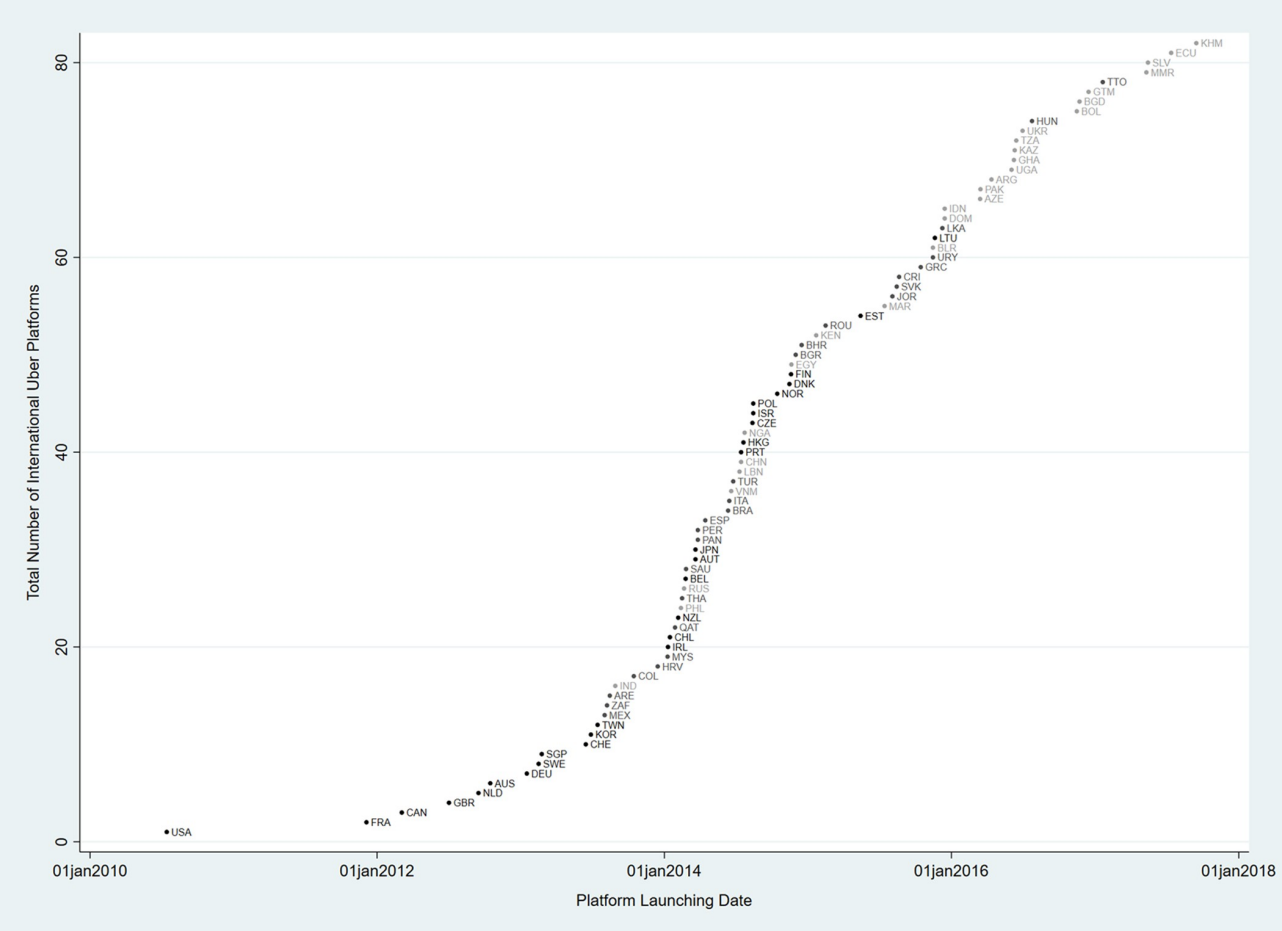
The international diffusion of Uber, 2010–2017.

As a pioneering sharing platform company, Uber has successfully stretched out to the global market [33]. Thus, Uber serves as a suitable case to fully capture the variations of institutional environments across national economies during the course of its global expansion. Founded in 2009, Uber first launched its online platform service connecting riders and drivers in July 2010 in San Francisco, U.S. After making its international debut in Paris, France, in 2011, Uber has rapidly diffused both domestically and internationally. Its platforms are now in operation over 700 cities in 80 different countries world-wide, standing out as the most valuable sharing economy organization followed by Airbnb. Fig 1 demonstrates the international diffusion of Uber platforms between 2010 and 2017. Darker color nodes indicate countries with high quality of legal-institutional environment, while brighter color nodes identify countries with low quality of legal-institutional environment.

Fig 1 shows a typical S-curve where the diffusion started at a slow pace, accelerated in the middle, and then slowed down toward the end. Actually, Uber has adopted a disruptive market strategy by launching its mobility platform service first and then calling for legislative permission from the government afterward [15]. Accordingly, its platform has been challenged by traditional actors after its launching in some countries. Since the focus of our study is to analyze the introduction of sharing economy on a global scale, however, we focus on the institutional factors that facilitate the international expansion of Uber, not on the institutionalization of its platforms afterwards.

In our effort to account for the expansion of Uber, we incorporate market uncertainty as the key concept in formulating our theoretical expectation. In the literature of organization studies, uncertainty is known to be a critical environmental factor in making strategic decisions [34–36]. Uncertainty in markets can be understood in terms of complexity, volatility, and randomness [37]. Low uncertainty in the institutional environment is likely to facilitate entrepreneurial activities of nascent firms such as Uber. Since Uber opens up its services without a stable legal status with the traditional economy, we expect that this platform company will make strategic decisions to launch platforms in states where it can minimize the already-high uncertainty. In other words, Uber is likely to choose nation-states with a stable legal and institutional environment in expanding its business.

Therefore, we expect that the legal and institutional environments will be critical in facilitating the diffusion of the sharing platform. Among legal-institutional environments, we particularly focus on two interrelated dimensions: (1) the establishment of the rule of law and (2) the quality of market regulation by the government. In states with a strong rule of law, Uber can minimize market uncertainty when it opens a platform and seeks for a stable legal status afterwards. Also, Uber can avoid inappropriate regulation by the government, sponsored by incumbent market players, if the nation-state does not arbitrarily regulate new entrepreneurial actors. Thus, we hypothesize as follows:

**Hypothesis 1:** Uber is more likely to launch its service in states with an established rule of law compared to states without rule of law.

**Hypothesis 2:** Uber is more likely to launch its service in states with a high quality of governmental regulations on markets compared to states with a low regulation quality.

## Data and methods

To test our hypothesis, we collected a unique cross-national dataset over the period from 2010 to 2017. Our data is based on Uber’s website where the company publicly posts the list of cities and countries where its platforms are in operation. As Uber does not disclose the exact launching dates for each city and country, however, we additionally collect the Uber launching dates for the first city of the corresponding countries by using related press releases and other news articles and by cross cross-checking the information with Uber’s website. The sources used for verification are shown in our deposited data file.

Our complete data consists of the first launching dates for the first expanded city in each country from 2010 to 2017. We measured those dates based on the exact timing when Uber made a decision to launch its platform to a given country. According to our data, there are 85 out of 187 countries where Uber service was expanded. The data is right-censored as of 2017 –the year when the diffusion curve has been flattened (please see Fig 1). We use the launching dates as the dependent variable where 1 indicates that Uber service is operating in a given country-year and 0 means that the service is not opened.

Corresponding to our main hypotheses on the effect of legal-institutional condition, we incorporate variables on legal-instutitional environments derived from the Worldwide Governance Indicators (WGI). WGI data composits a series of variables that capture multifaceted dimensions of governance of 215 countries betwen 1998 and 2018 [38]. The variables include (1) citizens’ voice and government-citizen accountability, (2) political stability and the absence of violence or terrorism, (2) governmental effectiveness, (4) regulatory quality of the government, (5) the establishment of the rule of law, and (6) governmental control of corruption [39]. Out of six pillars of the governance indicators, we selected two independent variables, the the rule of law and the quality of regulation, to operationalize legal and institutional conditions in our model. The rule of law variable captures the degree to which people display confidence in the law-abiding of society; the quality of regulation variable measures the government’s capacity to impose clear and predictable policies and regulations favoring private sector development [39]. These variables are normalized to a scale of -2.5 (bad) to 2.5 (good).

For a rigor test of our hypotheses, we control for a number of domestic factors. First of all, we incorporate the influence of the level of economic development and the basic demographical factors in our models. Using the Penn World Table 9.1 [40], we include the natural logged real GDP, expenditure size, population, and human capital (e.g., the years of schooling and the anticipated returns). From the World Development Indicatros (WDI) data of World Bank, we also include the percentage of urban population to total population [41].

Secondly, to control for the general political environment, we include the level of democracy and state fragility variables derived from the Polity IV dataset [42]. The level of democracy is measured by subtracting the institutionalized autocracy score from the institutionalized democracy score. Hence, the scale of the combined scores ranges from -10 (strongly autocratic) to 10 (strongly democratic). The state fragility variable denotes whether a polity is separated without an effective authority within the state border [42]. The variable is measured as a scale of 0 (no overt fragmentation) to 4 (serious fragmentation). Additionally, we incorporate the level of government expenditure, which his the percentage of consumption expenditure by the government vis-a-vis total domestic output from the WDI.

Lastly, we control for the industrial characteristics of countries, including the size of trade and the size of tourism, using data from the WDI. We also include unemployment rate and service sector employment in our dataset; information on employment is derived from the International Labor Organization Statistics (ILOSTAT) [43].

Our final dataset is collected from five data sources: Worldwide Governance Indicators (WGI) [38], World Development Indicators (WDI) [41] from World Bank, the Penn World Table 9.1 [40], the Polity IV dataset [42], and the International Labor Organization Statistics (ILOSTAT) [43]. Because of this combined nature of our data, the total number of N decreases as we add variables from different sources to each model. Hence, we tried to avoid ambiguity regarding data analysis by using listwise deletion. Table 1 shows the descriptive statistics of the dependent, independent, and control variables used in our empirical model.

**Table 1:** Descriptive statistics of independent variables.

	N	Mean	S.D.	Min.	Max.
Rule of Law	1,144	.015	1.003	-2.255	2.100
Quality of Regulation	1,144	.114	.966	-2.244	2.261
Real GDP (logged)	1,144	11.758	1.848	7.805	16.706
Human Capital	1,144	2.581	.691	1.166	3.974
Population (million)	1,144	48.920	161.015	.280	1409.520
Urban Population (%)	1,144	59.863	22.706	-.240	100
Level of Democracy	1,040	4.819	5.785	-10	10
State Fragility	1,040	.142	.561	0	3
Government Expenditure	1,040	15.800	5.157	-.310	37.885
Size of Trade (% of GDP)	637	88.616	58.028	-.200	416.389
Size of Tourism (% of exports)	637	10.098	9.665	-.001	50.663
Unemployment Rate	637	7.936	5.382	.389	27.695
Service Sector Employment	637	59.009	15.720	16.1	85.700

To analyze our longitudinal dataset, we employ event history analysis. As our observation of Uber expansion ends in 2017, years after 2017 are right-censored in the models. Event history analysis is appropriate when the focus of study is on the occurrence of a particular event over time. We particularly employ the Cox proportional hazards model, a semi-paramatric event history analysis model.

Cox regression is able to incorportate time varying multicovariates in a large sample analysis. In our models, we included multiple independent and control variables such as legal and institutional environments, economic and socio-demographical factors, political environments, and industrial characteristics. The total number of countries in the models is 142, with 77 countries experiencing the event of launching Uber service.

Unlike parametric models such as Weibull or Gompertz model, the Cox models do not assume any particular distribution for the duration time, leaving the baseline hazard rate nonparameterized [44]. It does not require any constraints on distributional assumptions, making it more attractive alternative to the parametric models. Cox regression in scalar form does not have an intercept term, for not assuming particular distribution for the duration time, as shown in Eq (1). Here, *t* denotes the duration time of survival which is the case of expansion of Uber in our analysis, and *h*(*t*) indicates the baseline hazard function. *h*_0_(*t*) represents the hazard rate at the time of *t* when all independent variables are set to zero. The coefficient estimates in this model show the incremental change of hazard, exp(*β*_*i*_), for a unit change of independent variables, *x*_*i*_. The general form of *h*(*t*) is further denoted in Eq (2).

$$h_{i}\left(t\right)=\text{exp}\left(\beta_{1}x_{1i}+\beta_{2}x_{2i}+\cdots +\beta_{k}x_{ki}\right)h_{o}\left(t\right)$$ (1)

$$h\left(t\right)=\underset{dt\to 0}{\text{lim}}P\left(t<T<t+dt|T>t\right)/dt$$ (2)

Our models passed the global test of proportional-hazards assumption on the basis of Schoenfeld residuals for Cox regression. We would not reject the null hypothesis that the hazards are proportional for our models. Table 2 illustrates the results of global tests for each model.

**Table 2:** Global tests of proportional-hazards assumption.

	chi2	df	Prob>chi2
Model 1	4.72	5	.451
Model 2	6.41	8	.601
Model 3	8.32	12	.760
Model 4	4.97	5	.420
Model 5	5.57	8	.695
Model 6	8.08	12	.779

Regarding multicollinearity problem, the correlation coefficient between the two main covariates, the rule of law and the quality of governmental regulation, is very high (0.928). To avoid any possibility of multicollinearity in our estimation, we test the effects of these institutional factors in separate models; model 1, 2, and 3, and model 4, 5, and 6. To provide additional information, we generated the correlation matrix of coefficients of Cox model. All analyses and computations used in our study were performed using Stata 16.0 MP.

## Results

How do the legal and institutional environments influence the international diffusion of Uber, the byword for sharing economy? We present our findings on the relationship between legal-institutional conditions and the establishment of Uber in Table 3.

**Table 3:** Hazard estimation for the international diffusion of Uber.

	Rule of Law			Quality of Regulation		
	Model 1	Model 2	Model 3	Model 4	Model 5	Model 6
Rule of Law	.671*** (.180)	.765*** (.197)	.620** (.233)			
Quality of Regulation				1.009*** (.216)	1.258*** (.254)	1.077** (.313)
Real GDP (logged)	.765*** (.100)	.704*** (.105)	.818*** (.172)	.851*** (.107)	.815*** (.114)	.813*** (.168)
Human Capital	.258 (.293)	.287 (.314)	.727 (.408)	-.031 (.305)	-.118 (.335)	.550 (.418)
Population (million)	-.001 (.001)	-.001 (.001)	-.001 (.001)	-.001 (.001)	-.001 (.001)	-.001 (.001)
Urban Population (%)	-.008 (.007)	.001 (.008)	-.002 (.009)	-.010 (.008)	-.001 (.009)	-.002 (.010)
Level of Democracy		-.006 (.024)	-.035 (.032)		-.032 (.025)	-.060 (.033)
State Fragility		.110 (.221)	.240 (.352)		.100 (.217)	.329 (.339)
Government Expenditure		-.065* (.033)	-.070 (.039)		-.047 (.031)	-.071 (.039)
Size of Trade (% of GDP)			.001 (.003)			-.001 (.003)
Size of Tourism (% of exports)			.048* (.019)			.038 (.020)
Unemployment Rate			.021 (.027)			.021 (.027)
Service Sector Employment			.005 (.010)			.006 (.010)
Log-likelihood	-301.856	-288.147	-188.739	-296.736	-281.711	-185.897
No. of observations	791	703	433	791	703	433
No. of failure	77	75	54	77	75	54
No. of country	142	129	88	142	129	88

^a^***p < .001,

**p < .01,

*p < .05 (two-tailed tests)

^b^Standard errors in parentheses.

To begin with, Models 1, 2, and 3 estimate the influence of an established rule of law on the international diffusion of Uber platforms. Results in Model 1 suggest that an established rule of law is positively and significantly associated with the hazard rate of launching an Uber platform by 1.956 times (= exp[0.671], p < 0.001), even after controlling for economic and demographic characteristics such as real GDP, the level of human capital, and the size of total and urban population. In Model 2, we additionally control for political environmental factors including the level of democracy, state fragility, and governmental expenditure, and the results show that a single unit increase in the rule of law variable is still positively associated with a 2.149 times increase (= exp[0.765]) in the hazard rate of opening the sharing service. In Model 3, which is the most saturated model with various industrial factors such as size of trade and tourism as well as unemployment rate and service sector employment controlled, our analysis still suggests that a stable and establiehd rule of law increases the rate of launching an Uber platform by 1.859 times (= exp[0.620], p < 0.001). The results from Models 1 to 3 clearly support hypothesis 1 on the critical role of legal institutions in facilitating the expansion of sharing economy platforms.

Among control variables, there were only two domestic factors that turn out to be significantly related to the opening of Uber services, which are real GDP and the size of tourism. Specifically, in the saturated model, one unit increase in the logged GDP score is positively and sigficantly associated with the rate of establishing an Uber service by 2.266 times (= exp[0.818], p < 0.001). In addition, the size of tourism in a given country is related to 6.9% increase (= exp[0.048], p < 0.05) in the rate of opening the service. The results implies that both higher economic output and larger size of tourism in an economy provide ample opportunities for this nascent mobility service to start its business. Other than these two control variables, other economic, political, and industrial factors do not have a statistically significant influence on the diffusion of Uber.

In Models 4, 5 and 6, we test the effect of regulation quality on the expansion of Uber platforms. The results suggest that the quality of governmental regulation is positively and significantly associated with the hazard rate of launching the sharing services by 2.743 times (= exp[1.009], p < 0.001) in Model 4, 3.518 times (= exp[1.258], p < 0.001) in Model 5, and 2.737 times (= exp[1.077], p < 0.01) in Model 6. These results provide empirical support for our expectation in hypothesis 2 that institutional environment matters for Uber’s expansion. When the government is capable of imposing clear and predictable policies and regulations in a given country, sharing econmy platforms are more likely to seek opportunities to strive. In addition, Models 4 to 6 show that Uber expands to countries with high level of GDP. As to the size of tourism in Model 6, however, the effect size decreases to the extent that the variable loses its statistical significance.

**Fig 2:**
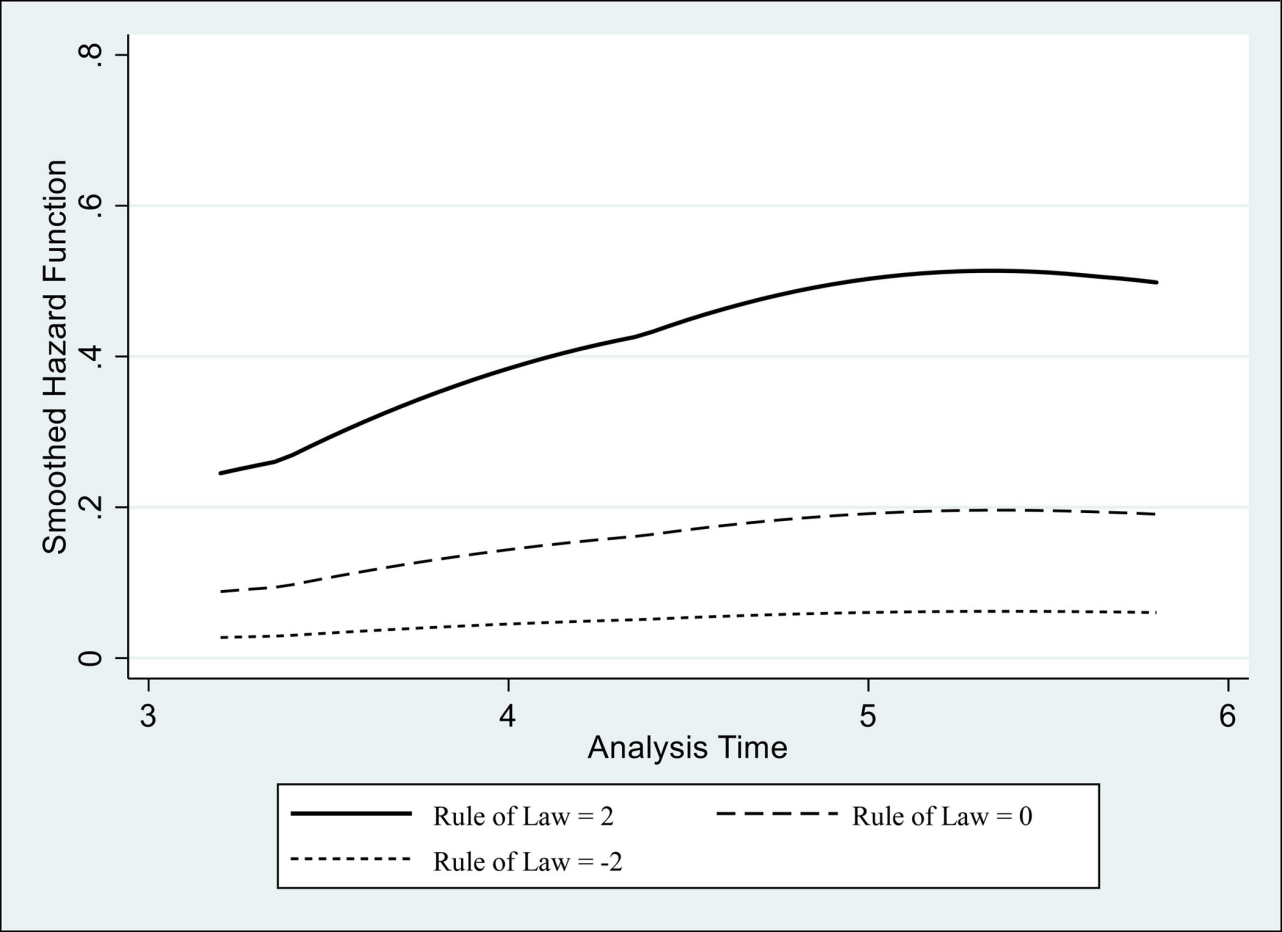
Rule of law and the hazard curves of the international diffusion of Uber. Higher Curve: Rule of Law = 2. Middle Curve: Rule of Law = 0. Lower Curve: Rule of Law = -2.

**Fig 3:**
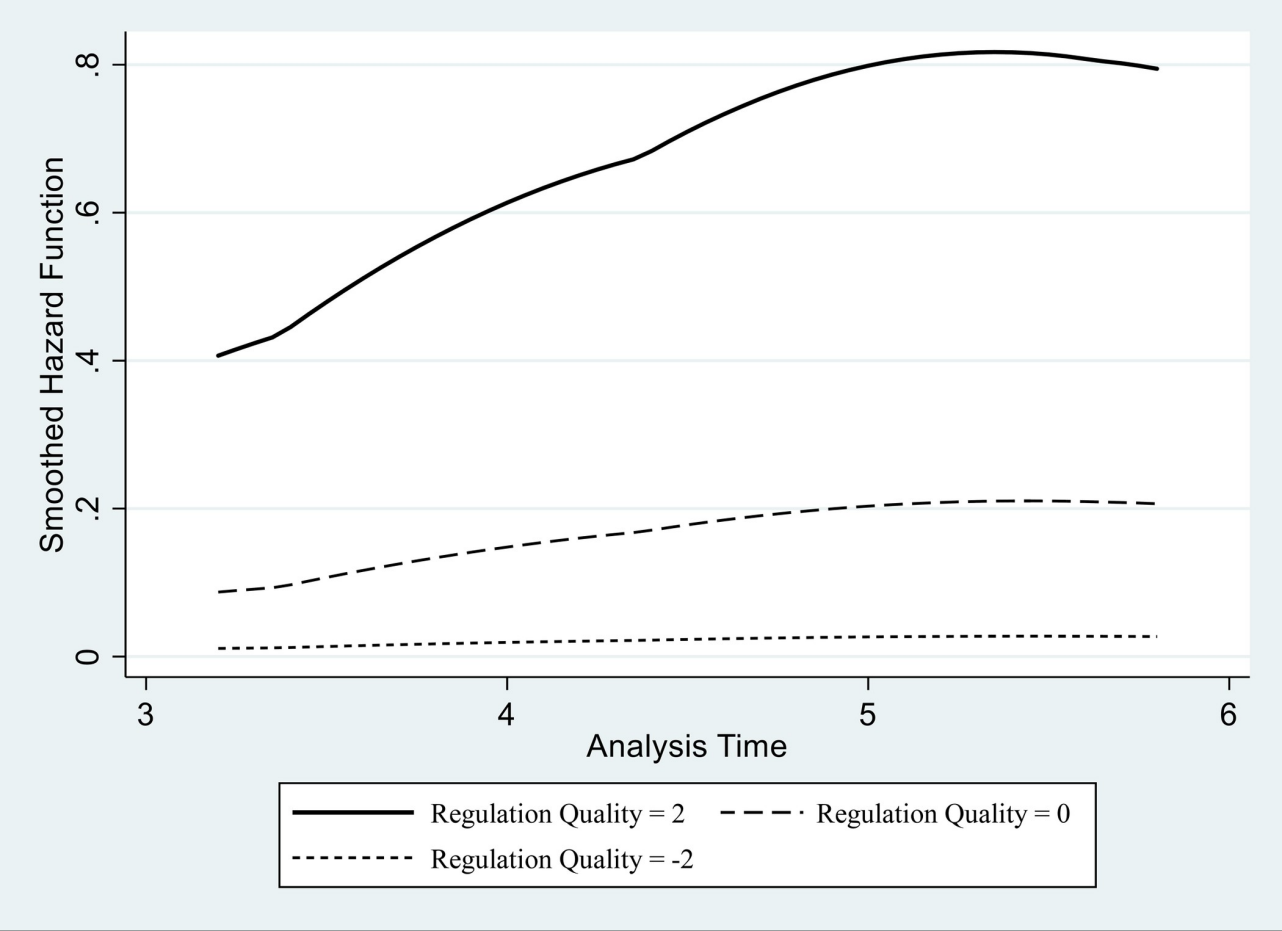
Regulation quality and the hazard curves of the international diffusion of Uber. Higher Curve: Regulation Quality = 2. Middle Curve: Regulation Quality = 0. Regulation Quality: Rule of Law = -2.

In Figs 2 and 3, we use Models 3 and 6, respectively, to visualize the hazard curves of the international diffusion of Uber platforms. These two figures present the changing hazard of launching Uber services, depending on the varying levels of the rule of law and regulation quality–when all other variables are held at their mean values.

Figs 2 and 3 demonstrate a clear positive effect of the legal-institutional environment on the hazard of launching Uber services. Regarding the rule of law, Fig 2 shows that Uber platforms are more likely to be founded in states with high scores in the rule of law variable. As to the quality of regulation, Fig 3 presents a more salient effect of the regulatory condition in launching the platform.

All in all, the results provide empirical evidence for our expectation that legal and institutional conditions matter for the spread of sharing economy. Our models support hypotheses 1 and 2 by showing that an established rule of law and a high quality of governmental regulation stand out, among any other factors, as the most important conditions for the sharing economy platform to expand.

While our statistical analyses show the general trend where legal-institutional quality is a main factor for the diffusion of Uber, as shown in Fig 1, it is true that Uber is also expanding in some countries with low quality of legal-institutional environment such as Pakistan (March of 2016) and Ecuador (July of 2017). However, these cases can still be explained in our models since Cox regression incorporates not only the occurrence of an event but also the length of time until the occurrence. Our model suggests that Uber is likely to be launched in countries with lower legal-institutional quality relatively late compared to the countries with higher legal-institutional quality. Still, there are exceptional cases such as India (August of 2013) and Philippines (February of 2014) where Uber expanded early but their legal-institutional quality is low. It would be important to scrutinize these exceptional cases with further qualitative inquiries. Notwithstanding the notable exceptions to the general and probabilistic pattern suggested by our analyses, we reiterate the importance of legal-institutional quality in the international diffusion of the sharing platform.

## Conclusion and discussion

As a rising industry in the network society, sharing economy has the potential to provide an alternative to, and even a substitute for, the traditional economic transaction. Among sharing platforms, we focused on the case of Uber, one of the online platforms that has successfully diffused internationally. Analyzing a longitudinal, cross-national dataset, we suggest that Uber is more likely to spread to nation-states under a stable legal-institutional environment. Taking the risk of launching its service prior to obtaining legal status, the sharing company aims to decrease its market uncertainty by expanding to states with an established rule of law or with predictable governmental regulations.

Our study has important contributions to the scholarship of sharing economy and entrepreneurship. First of all, our research identifies the main external environment under which sharing economy can flourish. To fulfill the potential of sharing platforms in facilitating peer-to-peer economic transactions and maximizing the use of products and assets, legal institutions such as the rule of law and the quality of governmental regulation turn out to be critical. States with a stable legal environment will be able to lead the sharing economy and to adopt alternative business models between suppliers and customers. In other words, sharing economy can strive under a certain quality of institutional design and regulatory regime. It is in line with the theoretical perspective that the sharing economy is not a mere emergent industry but rather a whole set of social relationships wherein people put the ethics of sharing into practice [21, 24–26]. Socio-demographical, economic, political, and industrial background failed to fully capture the international diffusion of the sharing platform. Mature institutional and legal arrangements are examined to be favorable to the new actors to move in and consolidate the ecosystem of sharing economy.

Secondly, and relatedly, our findings provide implication for the study of entrepreneurialship. Innovative startup firms such as Uber are destined to compete with conventional enterprises or incumbent industries in their effort to expand their services. Our study emphasizes the importance of legal-institutional environment in lowing the level of market uncertainty. We argue that, everything being equal, entrepreneurial firms which are embedded in a stable and predictable legal institutions will be more likely to develop and flourish compared to firms under an uncertain and unstable external environment.

Finally, our research suggests policy implications for encouraging entrepreneurship in national economies. Incumbent industrial actors are often hostile to disruptive innovators, and the existing regulatory frameworks are usually favorable to preexisting actors [17, 45]. If national and local governments revise their legal arrangements to minimize uncertainty that entrepreneurs encounter, the improved legal-institutional environment can serve as an incubater for new enterprises promoting alternative logics.

As with any research, our study has certain limitations. While we focus on the initial stage of diffusion when Uber expanded its mobility platforms since 2010, future studies are necessary to examine the consequence of Uber’s aggressive strategy to launch its service before receiving legal permission. With its disruptive market strategy, it is no wonder that Uber platform has been challenged by traditional actors after its launching in some countries [15]. In particular, the incumbent taxi industry has become a barrier for the sharing platform to successfully institutionalize itself as a legit service in various countries. A number of governments have even imposed either a partial or a full ban against Uber services. Thus, it would be important for the literature of sharing economy to wait and see whether and how Uber becomes accepted (or rejected) by the legislative and judicial bodies of a state in the future.

In addition, future studies are needed to explore the diffusion of sharing economy platforms beyond the case of Uber. Although Uber is one of the most important cases to study the international diffusion of sharing platforms, scholars of sharing economy may find different dynamics, either political or legal-institutional, in their study of other sharing services such as Airbnb, Lime, Lyft, Wikipedia, and others. Despite these limitation of our study, we expect that our study can open up future avenues for research on the relationship between legal-institutional environment and the rise of sharing economy.
